# Non-orbital Sclerosing Rhabdomyosarcoma Presented With Optic Neuropathy

**DOI:** 10.7759/cureus.21062

**Published:** 2022-01-09

**Authors:** Maizatul Nadia Hassan, Wan Hazabbah Wan Hitam, Nurul Ain Masnon, Chandran Nadarajan

**Affiliations:** 1 Department of Ophthalmology and Visual Science, School of Medical Sciences, Health Campus, Universiti Sains Malaysia, Kubang Kerian, MYS; 2 Department of Ophthalmology and Visual Science, Hospital Universiti Sains Malaysia, Kubang Kerian, MYS; 3 Department of Radiology, School of Medical Sciences, Health Campus, Universiti Sains Malaysia, Kubang Kerian, MYS; 4 Department of Radiology, Hospital Universiti Sains Malaysia, Kubang Kerian, MYS

**Keywords:** optic atrophy, facial rhabdomyosarcoma, parameningeal rhabdomyosarcoma, optic neuropathy, sclerosing rhabdomyosarcoma

## Abstract

Sclerosing rhabdomyosarcoma presentations are widely variable and non-specific initial features. We report a rare case of non-orbital sclerosing rhabdomyosarcoma presented with optic neuropathy. A 15-year-old female patient initially presented with upper gum swelling and pain for 3 months. It was associated with loosening of teeth. Subsequently, the patient developed recurrent epistaxis follow by left facial swelling and blurring of vision. Examination showed marked left facial swelling with mild proptosis. Visual acuity in the left eye was no perception of light with the presence of relative afferent pupillary defect. Fundoscopy showed left optic atrophy. Neuroimaging showed large aggressive soft tissue mass on the left infratemporal, masticator, and parapharyngeal space with a local extension to the sphenoid sinus. There was also an intracranial extension to the left temporal lobe with the base of skull bone destruction. Transnasal endoscopic biopsy revealed sclerosing rhabdomyosarcoma. Management was with chemotherapy. Sclerosing rhabdomyosarcoma may present with optic nerve involvement that may carry a guarded prognosis to the eyes.

## Introduction

Rhabdomyosarcoma (RMS) is an aggressive soft tissue sarcoma, a malignant neoplasm that is composed of cells with histopathologic features of striated muscle in various stages of embryogenesis. Sclerosing rhabdomyosarcoma (SRMS) is a rare subtype of RMS that was first described by Mentzel and Katenkamp in 2000 that is characterized by hyaline sclerosis and pseudovascular pattern [1]. Currently, there are only 42 cases of SRMS that had been described in the literature since it was initially reported [2]. We present a rare case of infratemporal, masticator, and parapharyngeal space SRMS with optic neuropathy in an adolescent female.

## Case presentation

A 15-year-old female patient presented with left-sided upper gum pain for three months. She was seen by a dentist and treated with antibiotics as an infected molar. She developed progressive loosening of teeth that worsened despite multiple dental treatments. In the following two months, she developed painful left facial swelling, nasal block, anosmia, and recurrent epistaxis. Her condition worsened and she started to have blurring of vision in the left eye. She went to the emergency department for treatment and was referred to the otorhinolaryngology team and ophthalmology team.

**Figure 1:**
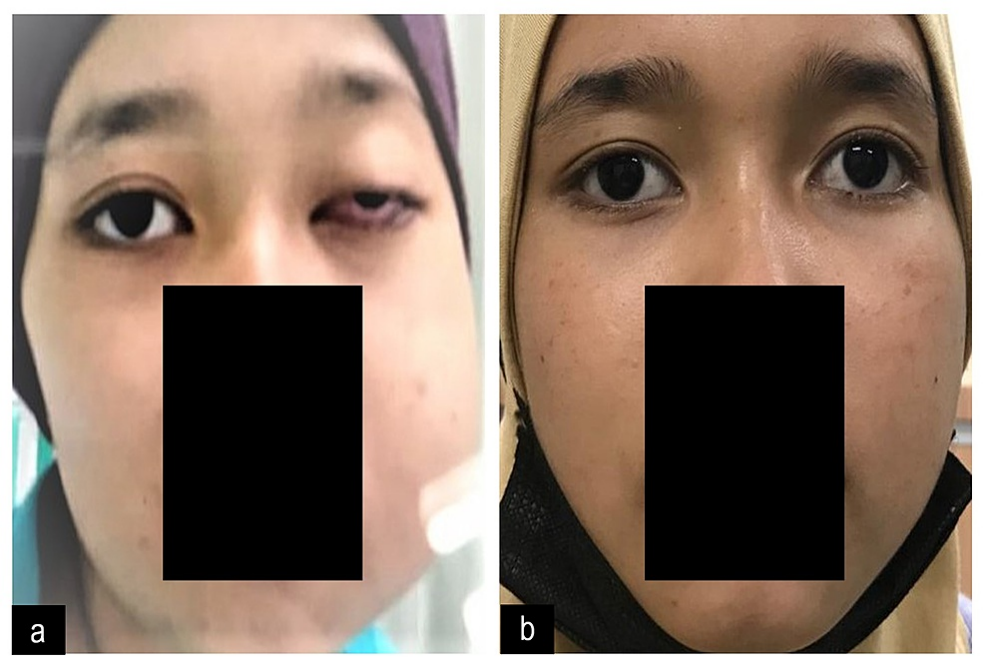
Pictures before and after starting treatment a) Left facial swelling extended from the periorbital region to the mandibular region with mechanical ptosis, b) Left facial swelling and ptosis improved after starting treatment.

Examination showed facial asymmetry with prominent left facial swelling extended from the periorbital region to the mandibular region (Figure 1). The swelling was firm and tender. There was reduced sensation over the left maxillary and mandibular region. Multiple lymph nodes were palpable in the left cervical region. Visual acuity in the left eye was no perception of light with the presence of relative afferent pupillary defect. The right visual acuity was 6/6. Extraocular movements were normal. There was mechanical ptosis in the left eyelid with injected conjunctiva. Other examinations of both anterior segments were unremarkable. Fundoscopy showed the presence of left optic atrophy. The right optic disc was pink and normal. Endoscopy examination by rhinologist showed the presence of mass occupying the whole left nostril. Neurological and other cranial nerves were normal. Systemic examination was unremarkable.

**Figure 2:**
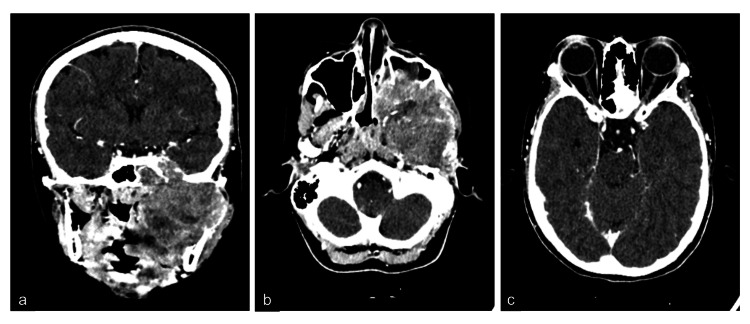
Contrast-Enhanced Computed Tomography (CECT) brain, orbit and paranasal sinus a) Coronal CT showed a significantly larger, ill-defined heterogeneous enhancing mass on the left side of the face. It occupied the left infratemporal, masticator, and parapharyngeal space with local and intracranial extension to the left temporal lobe. b) Axial CT showed bony destruction was observed at the base of the skull, lateral wall of the sphenoid, and left maxillary sinus. c) No invasion or compression of the optic nerve.

**Figure 3:**
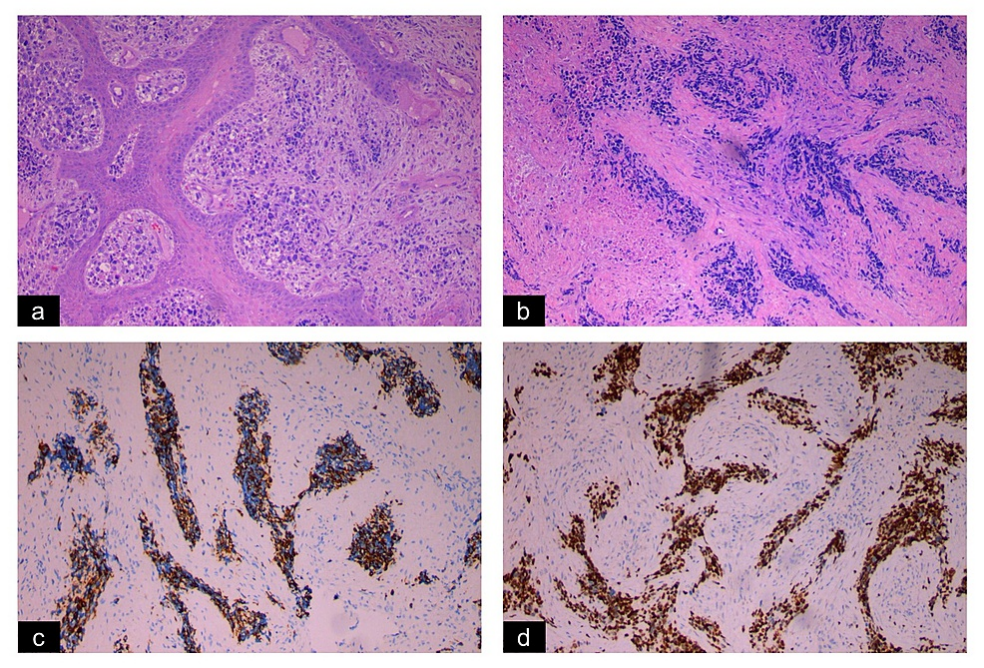
Histopathology and immunohistochemistry assessment of the mass a) Biopsy from the oral cavity (10x), hyperplastic squamous epithelium with round to oval pleomorphic eccentric nuclei and eosinophilic cytoplasm, b) Biopsy from nasal cavity (10x), fibrous/sclerosed tissue containing groups of pleomorphic tumour cells with marked crushed artifact, hyperchromatic nuclei, and eosinophilic cytoplasm. Immunohistochemical staining positive for c) Desmin (10x) and d) Myo D1 (10x).

Contrast-Enhanced Computed Tomography (CECT) brain and orbit revealed an ill-defined heterogeneous enhancing mass on the left side of the face. It occupied the left infratemporal, masticator and parapharyngeal space measuring 7.2 x 5.8 x 7.2 cm. There was a local and intracranial extension to the left temporal lobe. Bony destruction was observed at the base of the skull, lateral wall of the sphenoid and left maxillary sinus (Figure 2). Transnasal endoscopic biopsy revealed fragments of sclerosed tissue containing pleomorphic tumour cells with areas of tumour necrosis that stained positive for Desmin, CD56 and Myo D1. It was consistent with sclerosing rhabdomyosarcoma (Figure 3). There was no metastasis.

**Figure 4:**
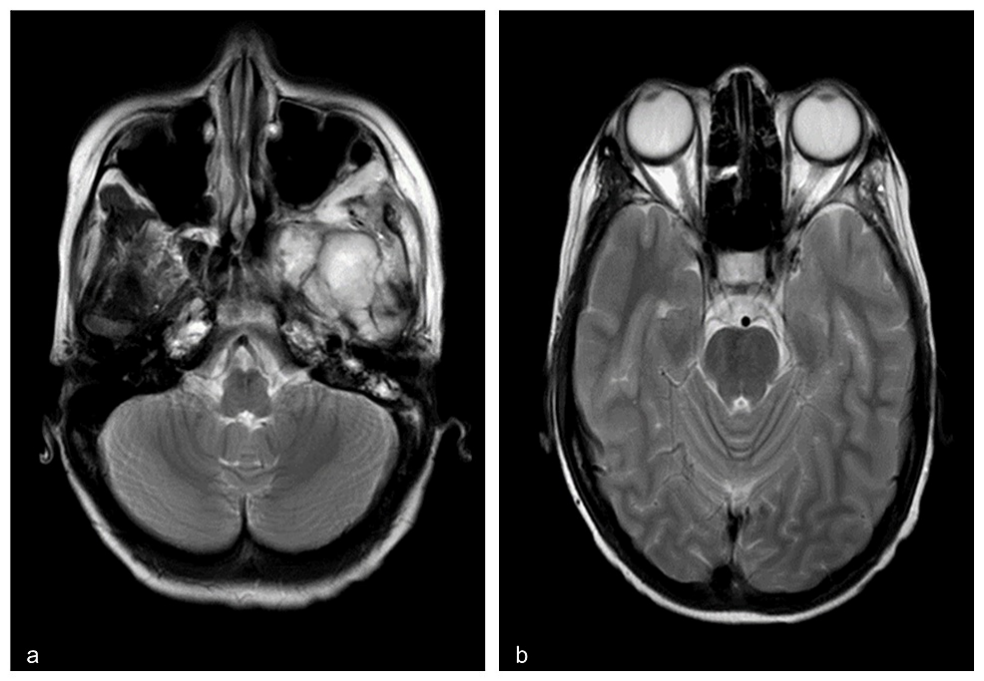
T2-weighted axial MRI brain a) Demonstrating heterogeneously hyperintense mass in left masticator space with locoregional involvement and extension, b) Demonstrating no enhancement of optic nerve and intruded of the tumour to posterior orbit.

The patient was treated with four cycles of IVADo chemotherapy (ifosfamide 3 g/m2, vincristine 1.5 mg/m2, actinomycin d 1.5 mg/m2, and doxorubicin 30 mg/m2), and six cycles of IVA (ifosfamide 3 g/m2, vincristine 1.5 mg/m2, and actinomycin d 750 mg/m2). Magnetic Resonance Imaging (MRI) of the brain and orbit was done after six months of chemotherapy showed that the tumour size had reduced, indicating a good response to treatment. There was no enhancement of the optic nerve or any extension to posterior orbit (Figure 4). However, the tumour was still too large for radiotherapy. Surgical intervention was not done because of the pterygoid muscle involvement at the base of the skull. The patient tolerated well with chemotherapy. Upon completion of treatment, her facial swelling improved significantly. The patient's visual acuity remained the same. Her condition remained stable following six months follow-up.

## Discussion

Sclerosing RMS (SRMS) presents at a wide age range (1-79 years) and has a bimodal age distribution, with a high number of patients identified in the first and second decades or around the fifth decade [3]. Males are 1.5 times more likely to develop SRMS in adolescence than females [2]. Our patient presented at age of 15 years old.

The most common symptoms of head and neck RMS tumors are facial swellings, dysphagia, nasal obstruction, epistaxis, and difficulty opening the mouth and speaking [4]. Persistent gum pain and progressive loosening of the teeth as seen in our patient have been reported but exceptionally rare. In a case series of advanced RMS with parameningeal and skull base invasion, Mullaney et al described sixth nerve palsy, followed by third, fifth, seventh, and fourth nerve palsies [5]. In addition to optic neuropathy, poor visual outcomes were related to orbital involvement, such as proptosis, ophthalmoplegia, tearing, or globe displacement owing to mass impact [6]. Our case is unique as she developed left eye optic neuropathy without other orbital symptoms except for mechanical ptosis due to left periorbital swelling in association with the left facial swelling.

Optic nerve infiltration may occur as a result of hematogenous spread, perineural invasion, or leptomeningeal metastatic dissemination as has been reported in earlier cases of leukemia, lymphoma, sarcoma, breast carcinoma, lung carcinoma, prostate cancer, pinealoblastoma, and rectal carcinoma [7-10].

Contrast-Enhanced Computed Tomography (CECT) and Magnetic Resonance Imaging (MRI) can provide crucial information in determining the size and location of the primary RMS tumor and its extension into adjacent tissues [11]. RMS appeared slightly hypodense or isodense with homogeneous enhancement in CT [12]. On T1-weighted images (T1WI), RMS showed isointensity and heterogeneous hyperintensity on T2-weighted images (T2WI) [13]. A previous study showed that heterogeneous hyperintensity on T2WI and nodular-shaped enhancement patterns in tumors may be considered as specific MRI features for RMS [12]. Immunohistochemistry showed RMS positive to skeletal muscle markers including myogenin, desmin, sarcomeric actin, and myoglobin [14]. Although meningeal and optic nerve enhancement may be detected to indicate optic nerve infiltration, literature has shown that negative neuroimaging does not rule out infiltration [15]. Previous studies showed limitations of MRI and CECT in reliably predicting prelaminar, laminar, or post laminar infiltration of the optic nerve, thus histopathologic analysis should be done to confirm optic nerve infiltration [16,17]. Our case is unique as she developed left eye optic neuropathy without any evidence of optic nerve involvement in neuroimaging.

The International Rhabdomyosarcoma Study Group (IRSG) has been leading protocols to help define the shifting therapy paradigms for RMS since 1972. RMS is usually treated with multimodality therapy combines chemotherapy with surgery and/or radiotherapy [18]. In our case, the surgical approach was avoided since complete surgical resection is a challenging procedure and generally impossible. Potential severe permanent functional dysfunction, cosmetic deficits, and morbidities such as cranial nerve paralysis or reduced temporomandibular joint motility have been observed in prior studies following surgical excision [18,19]. RMS is chemosensitive as well as radiosensitive. Radiotherapy can be postponed until week 13 without jeopardizing clinical outcomes [20].

The overall survival of 16 pediatric cases of SRMS with a short follow-up of <2 years was found to be 87.5% and disease-free survival was 62.5%. But the incidence of pediatric SRMS treatment failure was significantly high at 43.75% [2]. In 1995, the International Classification of Rhabdomyosarcoma (ICR) has provided a prognostically relevant classification system according to histology subtypes. Botryoid and spindle cell RMS give a superior prognosis, embryonal RMS has an intermediate prognosis, and alveolar RMS leads to an unfavorable prognosis. The fourth edition of the World Health Organisation (WHO) Classification of Tumors of Soft Tissue and Bone has updated the classification of RMS into alveolar (ARMS), embryonal (ERMS), botryoid RMS, spindle cell or sclerosing (SRMS), and pleomorphic (PRMS) subtypes of RMS. Optic neuropathy could be a potential complication of non-orbital RMS. Optic nerve infiltration may be difficult to be detected without a complaint from the patient and in the absence of radiological findings. Early ophthalmological referral and follow-up may contribute to favorable outcomes.

## Conclusions

Early optic neuropathy may be a sign of head and neck SRMS, and non-specific symptoms may cause a delay in diagnosis. Fortunately, treatment outcome with chemotherapy is promising whereas radiotherapy is more challenging due to its size. Hence the need for a multidisciplinary approach in managing SRMS is of paramount importance.
